# Synthesis of Pyrroloquinones via a CAN Mediated Oxidative Free Radical Reaction of 1,3-Dicarbonyl Compounds with Aminoquinones

**DOI:** 10.1155/2013/262580

**Published:** 2013

**Authors:** Thao Nguyen, Dwayaja Nadkarni, Shilpa Dutta, Su Xu, Sanghun Kim, Srinivasan Murugesan, Sadanandan Velu

**Affiliations:** 1 Department of Chemistry, University of Alabama at Birmingham, 901 14th Street South, Birmingham, AL 35294-1240, USA; 2 Center for Biophysical Sciences and Engineering, University of Alabama at Birmingham, 901 14th Street South, Birmingham, AL 35294-1240, USA; 3 Comprehensive Cancer Center, University of Alabama at Birmingham, 901 14th Street South, Birmingham, AL 35294-1240, USA

## Abstract

Pyrroloquinone ring systems are important structural units present in many biologically active molecules including a number of marine alkaloids. For example, they are found in a series of marine metabolites, such as tsitsikammamines, zyzzyanones, wakayin, and terreusinone. Several of these alkaloids have exhibited antimicrobial, antimalarial, antifungal, antitumor, and photoprotecting activities. Synthesis of pyrroloquinone unit is the key step in the synthesis of many of these important organic molecules. Here, we present a ceric (IV) ammonium nitrate (CAN) mediated oxidative free radical cyclization reaction of 1,3-dicarbonyl compounds with aminoquinones as a facile methodology for making various substituted pyrroloquinones. 1,3-dicarbonyl compounds used in this study are ethyl acetoacetate, acetylacetone, benzoyl acetone, and *N,N*-dimethyl acetoacetamide. The aminoquinones used in this study are 2-(benzylamino)naphthalene-1,4-dione and 6-(benzylamino)-1-tosyl-1*H*-indole-4,7-dione. The yields of the synthesized pyrroloquinones ranged from 23–91%.

## 1. Introduction

**Figure 1:**
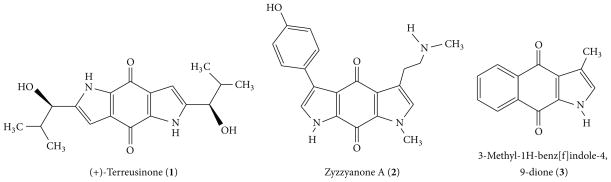
Selected natural products containing pyrroloquinone units.

Pyrroloquinone is a pharmacophore present in many biologically important molecules. For example, a family of marine alkaloids that include zyzzyanones, tsitsikammamine, and wakayin contains the pyrroloquinone skeleton [1–4]. These alkaloids are metabolites of marine sponges *Latrunculia*, *Zyzzya* and ascidian species *Clavelina* [1, 4–6]. In addition to antimicrobial, antimalarial, and antifungal activities, these alkaloids have notable antitumor properties, which are derived from their unique fused ring structure. Tsitsikammamine A derivatives, for example, inhibit indoleamine-2,3-dioxygenase, an important enzyme contributing to tumor immune invasion [7]. Tsitsikammamines A-B also exhibit antimicrobial, antimalarial, and antifungal properties, cytotoxicity, and topoisomerase inhibition [8]. Additionally, marine alkaloid wakayin inhibits topoisomerase II, damages DNA, exhibits strong antimicrobial property against *Bacillus subtilis,* and is potent toward human colon cancer cell lines [1]. Zyzzyanones A-D contain a bispyrroloquinone ring system and have exhibited cytotoxicity against Ehrlich carcinoma cells at micromolar range [2, 3]. Moreover, bispyrroloquinone is also the core structure of the marine fungus metabolite terreusinone (**1**), which is a potent UV-A protectant [9]. The photoprotecting activity of terreusinone is stronger than that of the commercial sunscreen ingredient oxybenzone [10]. For these reasons, pyrroloquinone alkaloids are regarded as a source of new antitumor and dermatological drugs [11–17]. In addition to this, 3-methyl-1H-benz[f] indole-4,9-dione (**3**) is a natural product isolated from the barks of *G. tapis* and *G. uvaroides* containing pyrrolonaphthaquinone ring system. This compound shows strong inhibitory effects on platelet-activating factor (PAF) receptor [18]. Selected few examples of the natural products containing pyrroloquinone units are given in Figure 1.

Unfortunately, these natural products are isolated from natural sources only in minute quantities, which impose a limitation on their thorough biological evaluation. Additionally, the unique fused-ring aromatic structure poses a challenge in the total synthesis of these natural products. Several efforts have been made towards achieving the synthesis of these natural products [8, 10, 17, 19]. Our group has recently used an oxidative free radical cyclization reaction as a key step in the synthesis of zyzzyanones and the intermediates [19, 20].

Oxidative free radical reactions facilitated by transition metals have been known to promote carbon-carbon bond formation. In these reactions, the electron transfer between the radical precursor and metal complex generates electrophilic radicals, which ultimately react with alkenes, alkynes, or quinones to form carbon-carbon bonds [21–26]. Among the metal salts that have been investigated in the past two decades for facilitating oxidative free radical cyclization, Mn-(OAc)_3_ and ceric (IV) ammonium nitrate (CAN) were proven to be the most efficient catalysts. A proposed mechanism of action for these reagents for effecting oxidative free radical cyclization has also been reported [21, 27, 28]. Mn-(OAc)_3_ and CAN have been extensively used in the synthesis of naphthoquinone, which is an important skeleton of natural products, such as mitosenes, kinamycins, and murrayaquinones [29, 30]. The synthesis of bispyrroloquinone by a CAN mediated oxidative free radical cyclization has been reported from our lab [20]. An Mn (OAc)_3_ mediated oxidative free radical cyclization leading to the total synthesis of zyzzyanones A-D has also been reported from our lab [19]. As an extension of these studies, herein, we demonstrate the general synthetic utility of CAN mediated oxidative free radical cyclization of various aminoquinones with 1,3-dicarbonyl compounds to form 24 new substituted pyrroloquinones.

## 2. Results and Discussion

The oxidative free radical reaction leading to the formation of pyrroloquinones is shown in the general Table 1. Substituted benzylaminoquinones such as 2-(benzylamino)naphthalene-1,4-dione (**8**) and 6-(benzylamino)-1-tosyl-1*H*-indole-4,7-dione (**10**) are treated with various 1,3-dicarbonyl compounds in the presence of CAN to afford 24 pyrroloquinone derivatives (**4a-l** and **5a-l**) inmoderate to excellent yields.

**Scheme 1:**
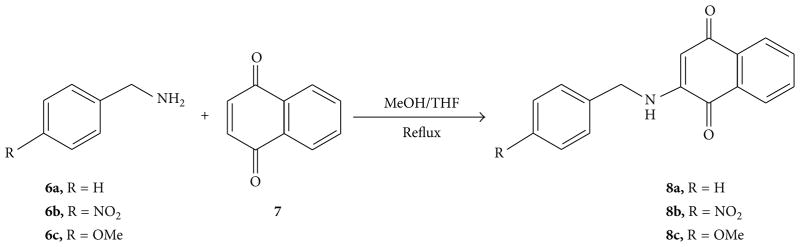
Synthesis of 2-benzylaminonaphthalene-1,4-diones.

We used various 2-benzylaminonaphthalene-1,4-diones (**8a–c**) and N-Tosyl-6-benzylaminoindole-4,7-quinones (**10a–c**) as starting substrates for our oxidative free radical reactions yielding pyrroloquinones. Three 2-benzylamino naphthalene-1,4-diones (**8a–c**) used in these studies were prepared as outlined in Scheme 1. Naphthalene-1,4-dione (**7**) was treated with substituted benzyl amines (**6a–c**) in a mixture of MeOH and THF to afford 2-benzylamino naphthalene-1,4-diones (**8a–c**) in 50–86% yield.

**Scheme 2:**
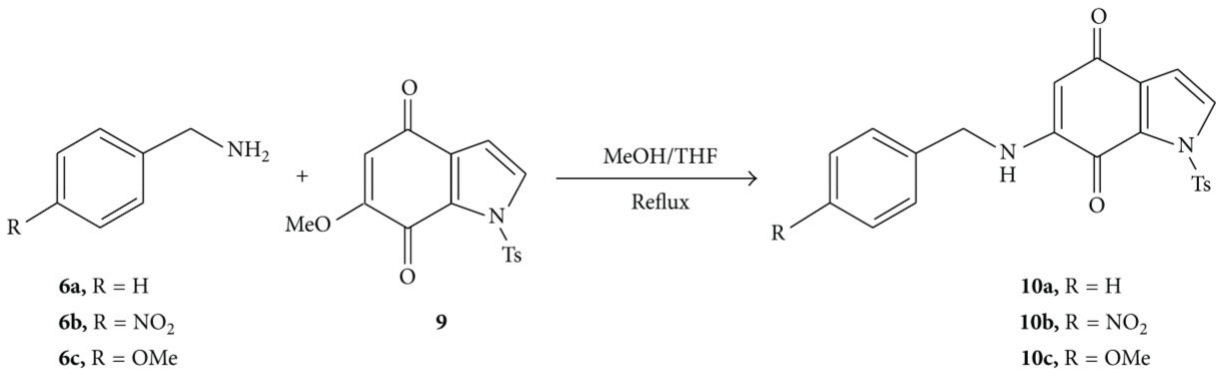
Synthesis of 6-(benzylamino)-1-tosyl-1H-indole-4,7-quinones.

The three N-Tosyl-6-benzylaminoindole-4,7-quinones (**10a–c**) used in these studies were prepared as outlined in Scheme 2. Previously reported [31], N-Tosyl-6-methoxyindole-4,7-quinone (**9**) was treated with substituted benzyl amines (**6a–c**) in a mixture of MeOH and THF to afford N-Tosyl-6-benzylaminoindole-4,7-quinones (**10a–c**) in 70–94% yield.

Yields and the specific reaction conditions used for the oxidative free radical cyclization of the aminoquinones (**8a–c** and **10a–c**) with four 1,3-dicarbonyl compounds are summarized in Figure 4. 1,3-dicarbonyl compounds used in this study are ethyl acetoacetate, acetylacetone, benzoyl acetone and *N*,*N*-dimethyl acetoacetamide. The reactions resulted in the formation of 24 new substituted pyrroloquinones with yields ranging from 23% to 91%. Most of these reactions were carried out in CH_2_Cl_2_ and MeOH, except for entries 2 and 3 where a combination of CH_2_Cl_2_ and EtOH was used as solvent. Initially, the reaction of **8a** with 4 equivalents of ethyl acetoacetate yielded the expected product **4a** in good yield (80%, entry 1). However, latter reactions employed fewer equivalents of *β*-carbonyl compounds, which tended to result in cleaner reactions and made purification easier. Unfortunately, when compound **8b** reacted with ethyl acetoacetate in CH_2_Cl_2_ and MeOH, the reaction proceeded well, but a mixture of methyl and ethyl esters was obtained due to transesterification. So, a combination of CH_2_Cl_2_ and EtOH was used in these cases to obtain the products, **4b** and **4c** in 35% and 82%, respectively (entries 2 and 3).

The majority of pyrroloquinones **4a-l** were obtained in good yields regardless of the types of *β*-carbonyl starting materials used. The products **4a-l** were usually yellow compared to the red orange starting materials **8a–c**. Additionally, we experimented with 1, 1.8, and 4 equivalents of *β*-carbonyl compounds and found that only 1 equivalent of *β*-carbonyl compounds was sufficient to bring the reaction to the completion. Highest yields were obtained when the electron donating methoxy group was present on the benzyl substituent (entries 3, 6, 9, and 12, 82–91% yield). In contrast, the presence of nitro group on the benzyl group resulted in significantly lower yields (entries 2, 5, 8, and 11, 31–38% yield). Entries with unsubstituted benzyl substituents resulted in moderate yields (entries 1, 4, 7, and 10, 51–80%). The reactions of **8b** with all four 1,3-dicarbonyl compounds proved to be difficult. This is due to several reasons, firstly, poor solubility of **8b** in CH_2_Cl_2_ and MeOH which required the usage of triple amount of solvent volume and heating to dissolve the starting material. Secondly, the reaction did not proceed at room temperature as in the other cases and needed refluxing to force the reactions to go to completion. In addition, more CAN (1.5–2.5 equiv) and longer reaction times were required for the reaction to go to completion. Finally, the reactions always resulted in a significant amount of side products, which ultimately led to low product yields.

**Figure 2:**
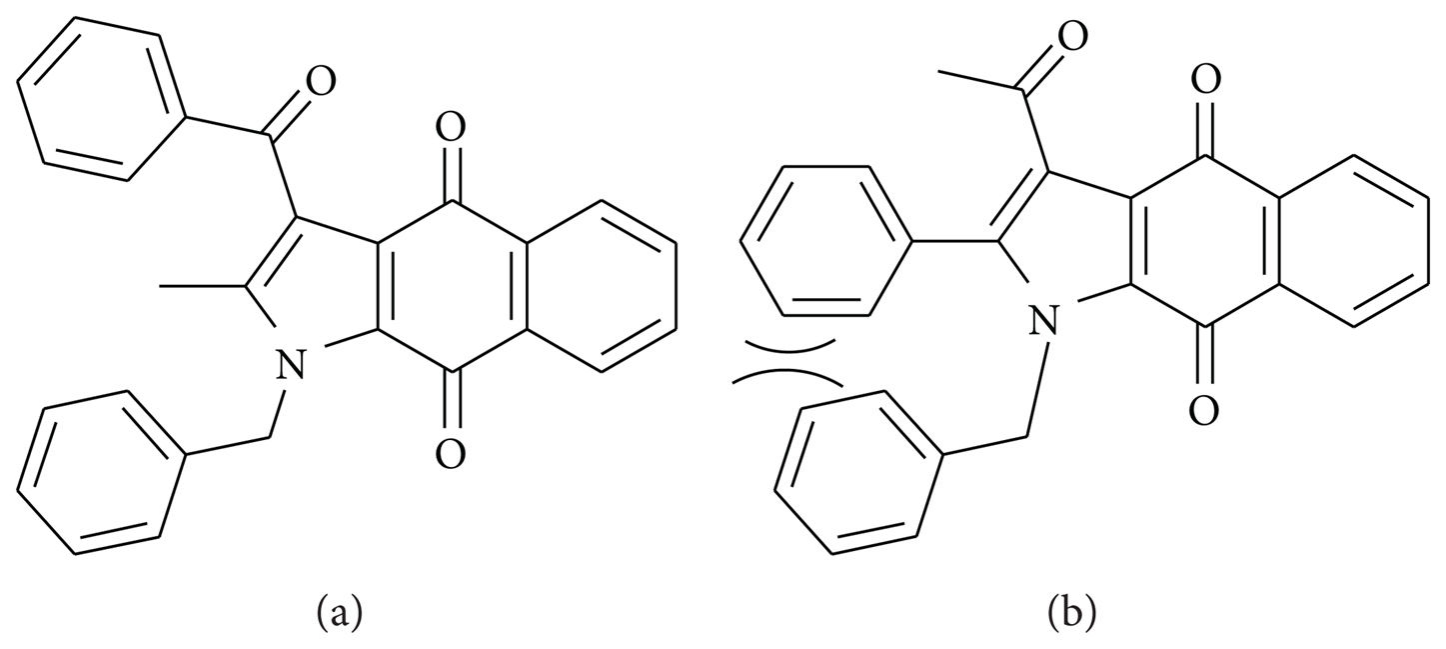
Two possible regioisomers of compound **4g**.

**Figure 3:**
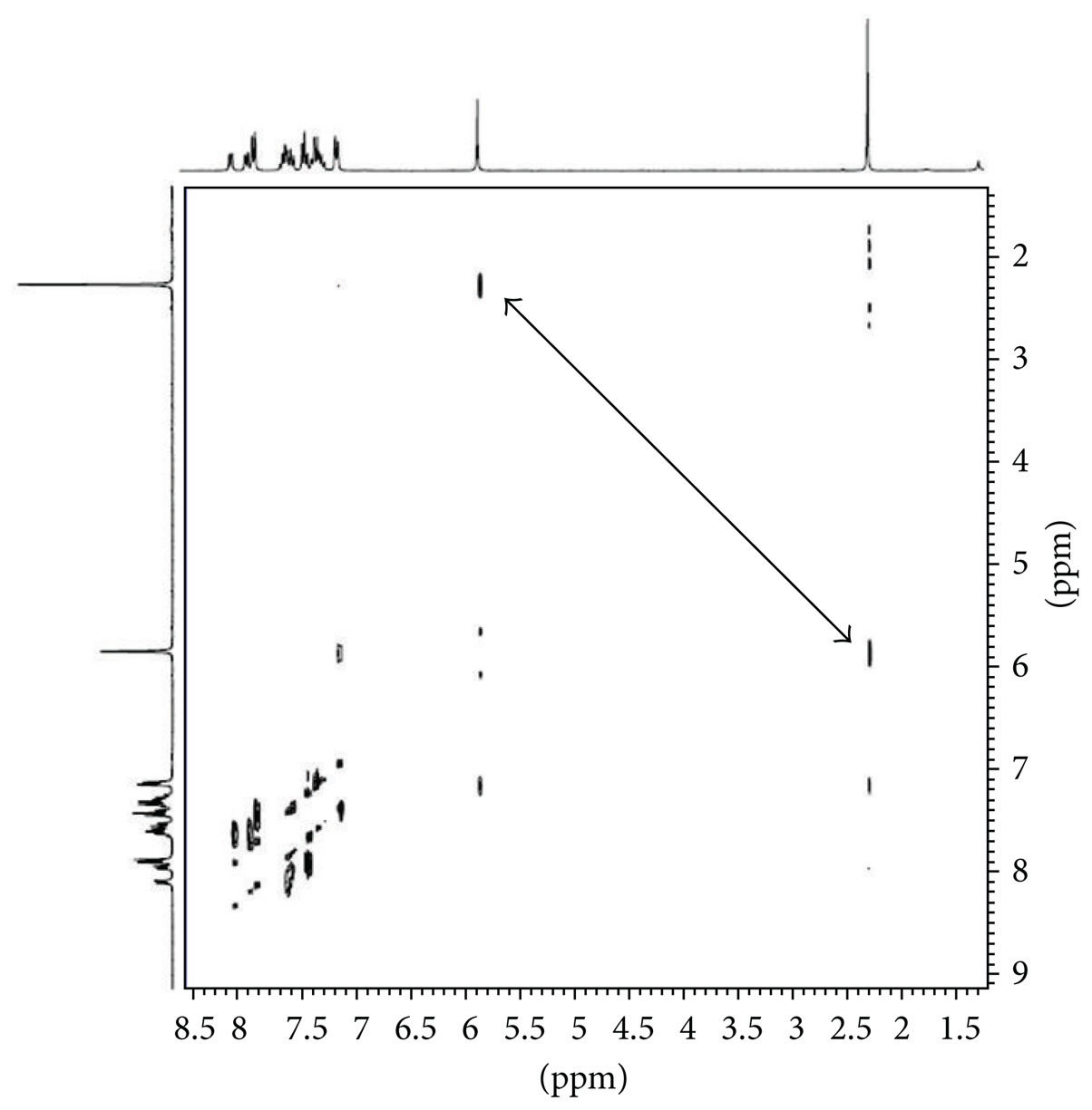
NOESY spectrum of compound **4g**.

When 1,3-diketones such as acetylacetone and benzoylacetone are used in these experiments, theoretically, two regioisomeric products are possible. The two possible products (A and B) for the reaction between benzoylacetone and 2-benzylamino naphthalene-1,4-dione mediated by CAN are illustrated in Figure 2. However, only one product (**4g**) was formed in this reaction, and it was proved to be the isomer A by ^1^H-NMR, ^13^C-NMR, and NOESY experiments. In the NOESY NMR experiment of compound **4g** as indicated in Figure 3, the methyl group (CH_3_; singlet; 2.18ppm) clearly had a NOESY correlation with the benzyl methylene group (CH_2_; singlet; 5.76 ppm). The experiment clearly establishes that the product formed is regioisomer **A**. The absence of the regioisomer **B** is perhaps due to the steric hindrance between the two bulky phenyl groups, which makes the structure significantly more unstable.

**Figure 4:**
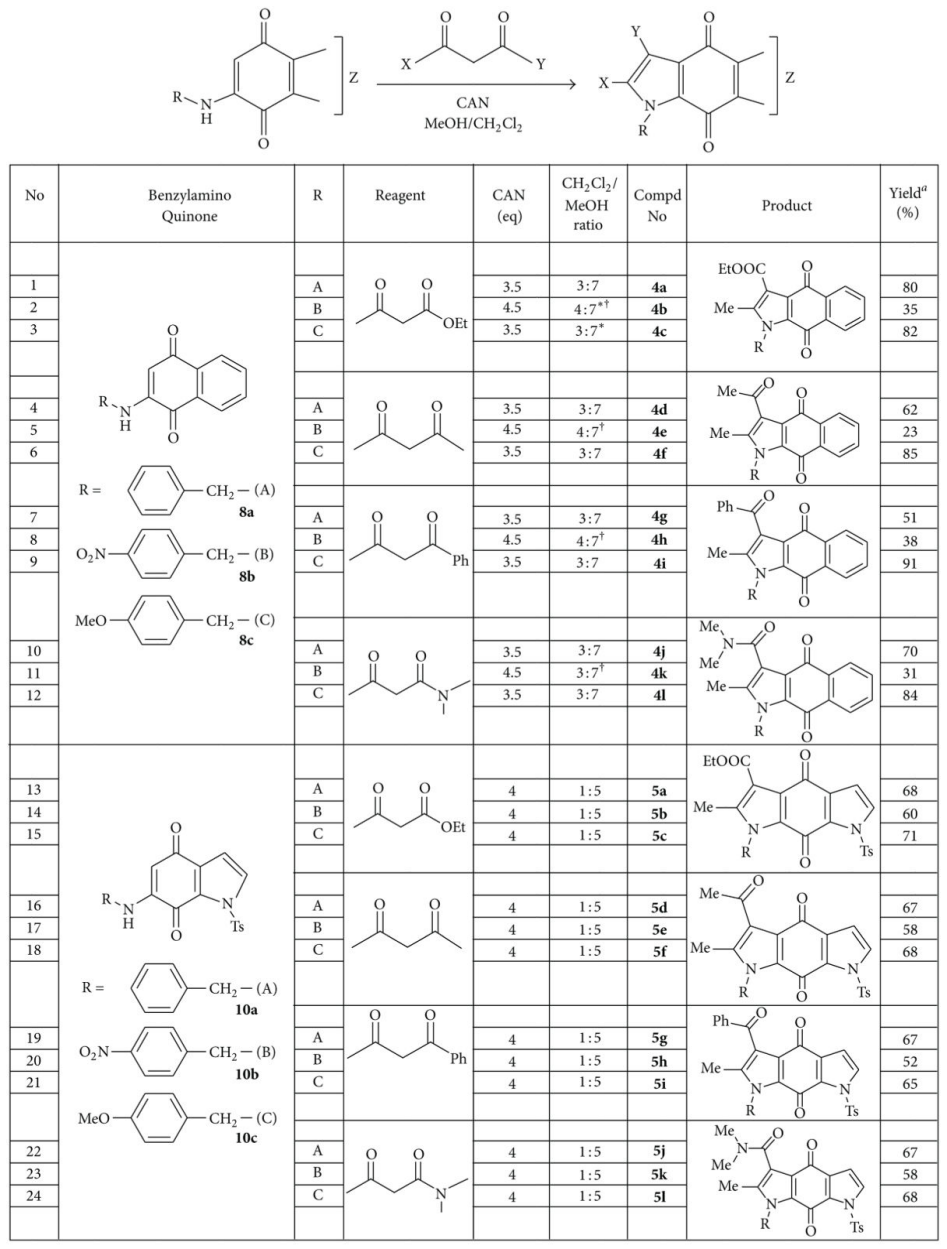
CAN-mediated oxidative free radical cyclization reaction of benzylamino quinones yielding substituted N-benzyl pyrroloquinones. ^a^isolated yields; ^*^EtOH/CH_2_Cl_2_ mixture was used instead of MeOH/CH_2_Cl_2_ to avoid transesterification; ^†^triple volume of solvents and heating was used to dissolve starting materials.

Compounds **10a–c** were reacted with the 1,3-dicarbonyl reagents, including ethyl acetoacetate, benzoyl acetone, *N*,*N-*dimethyl acetoacetamide, and acetylacetone. The reactions were carried out in the 1 : 5 ratio mixtures of CH_2_Cl_2_ and MeOH. The yields and reaction condition to obtain the final products **5a-l** are given in Figure 4. In this study, four equivalents of CAN were necessary for the reactions to complete while less equivalents or absence of CAN resulted in the incomplete or no reactions. The types of 1,3-dicarbonyl reagents did not affect the outcome of the reaction as there were no trends affecting percent yields when different *β*-carbonyl reagents were used. Interestingly, the reaction of 6-(benzylamino)-1-tosyl-1*H*-indole-4,7-dione with 1,3-diketones is expected to yield two regioisomeric products, but only one product was formed as in the case of 2-(benzylamino)naphthalene-1,4-dione system.

Although the trend is not as strong as in previous 2-(benzylamino)naphthalene-1,4-dione system, the yields of the methoxybenzyl-substituted products **5c, f, i,** and **l** (entries 15, 18, 21, and 24, 65–71%) are slightly higher than those of the nonsubstituted counterparts **5a, d, g,** and **j** (entries 13, 16, 19, and 22, 67–68%). In addition, the nonsubstituted bispyrroloquinones **5a, d, g,** and **j** were achieved in better yields compared to their equivalent nitrobenzyl substituted compounds **5b, e, h,** and **k** (entries 14, 17, 20, and 23, 52–60%). These results were consistent with our earlier observation in the reactions of 2-benzylaminonaphthalene-1,4-diones.

## 3. Conclusions

Synthesis of pyrroloquinone unit is the key step in the synthesis of several biologically important organic molecules. A CAN mediated oxidative free radical cyclization reaction of 1,3-dicarbonyl compounds with aminoquinones leading to the formation of various substituted pyrroloquinones is presented. 1,3-dicarbonyl compounds used in this study are acetylacetone, benzoyl acetone, ethyl acetoacetate, and *N*,*N*-dimethyl acetoacetamide. The aminoquinones used in this study are 2-(benzylamino)naphthalene-1,4-dione and 6-(benzylamino)-1-tosyl-1*H*-indole-4,7-dione. The yields of the synthesized pyrroloquinones ranged from 23 to 91%. Interestingly, we found that only one regioisomer was formed even when 1,3-diketones like benzoyl acetone were used. Finally, the majority of the oxidative free radical cyclized products were isolated as yellow solids in good yields.

## 4. Experimental

### 4.1. General Considerations

The NMR spectra were recorded on a Bruker DPX 300, DRX 400, or AVANCE 700 spectrometers. Chemical shifts are reported in ppm relative to TMS or CDCl_3_ as internal standard. The values of chemical shift (*δ*) and coupling constants *J* were given in parts per million and in Hz, respectively. Mass spectra were recorded using an Applied Biosystems 4000Q Trap and Micromass Platform LCC instruments. Thin-layer chromatography was performed with silica gel plates with fluorescent indicator (Whatman, silica gel, UV254, and 25 *μ*m plates) and visualized by UV (wavelengths 254 and 365 nm). The reaction mixture was purified by column chromatography using silica gel (32–63 *μ*m) from Dynamic Absorbent Inc. Melting points were uncorrected and obtained from Mel-Temp II apparatus. Solvents were removed *in vacuo* by using rotatory evaporator. The recrystallization was assisted by Fisher Scientific FS30 sonicator. Anhydrous solvents were purchased in Sure-Seal bottles from Aldrich chemical company. Other reagents were obtained from Aldrich and Acros chemical companies.

#### 4.1.1. 2-(Benzylamino)naphthalene-1,4-dione (8a)

To a solution of 1,4-naphthoquinone **7** (5.0 g, 31.62mmol) in THF (50mL), a solution of benzylamine **6a** (6.91 mL, 63mmol) in MeOH (50 mL) was added. The reaction was refluxed under N_2_ atm for 36 h. Upon the completion of the reaction as indicated by TLC (100% CH_2_Cl_2_), the solvents were removed *in vacuo*. The residue obtained was dissolved in EtOAc (700 mL) and washed with water (2 × 200 mL), brine (200 mL) and dried over Na_2_SO_4_. The drying agent was filtered off, and the solution was concentrated under reduced pressure to obtain the crude product which was then purified by chromatography over silica gel using 100% CH_2_Cl_2_ as eluent to afford compound **8a** as a red solid (6.5 g, 80%); Mp: 137–141°C; ^1^H NMR (CDCl_3_, 300MHz) *δ* 4.37 (d, 2H, *J* = 5.7Hz), 5.78 (s, 1H), 6.26 (bs, 1H), 7.25–7.44 (m, 5H), 7.61 (t, 1H, *J* = 7.5Hz), 7.73 (t, 1H, *J* = 7.5Hz), and 8.01–8.12 (m, 2H); ^13^C NMR (CDCl_3_, 75MHz) *δ* 46.9, 101.9, 126.3, 126.4, 127.8 (2C), 128.3, 129.2 (2C), 130.6, 132.2, 133.7, 134.9, 136.1, 147.9, 182.0, and 183.2; MS (ES+): *m/z* = 264 [M+ H].

#### 4.1.2. 2-(4-Nitrobenzylamino)naphthalene-1,4-dione (8b)

To a solution of 1,4-naphthoquinone **7** (0.30 g, 1.90mmol) in THF (7 mL), a mixture of 4-nitrobenzylamine hydrochloride **6b** (0.54 g, 2.84mmol) and Et_3_N (0.383 g, 3.79mmol) in MeOH and CH_2_Cl_2_ (1 : 1, 14 mL) was added. The reaction was stirred under N_2_ atm overnight at room temperature. After the TLC analysis (EtOAc/hexanes, 1 : 2) showed the completion of the reaction, the solvents were removed *in vacuo*. The residue obtained was dissolved in CH_2_Cl_2_ (300 mL) and washed with water (2 × 100 mL), brine (100 mL) and dried overNa_2_SO_4_. The drying agent was filtered off, and the filtrate was concentrated under reduced pressure to obtain the crude product which was purified by chromatography over silica gel using 100% CH_2_Cl_2_ as eluent to afford compound **8b** as a red solid (0.292 g, 50%); Mp: 225–228°C; ^1^H NMR (CDCl_3_, 300MHz) *δ* 4.54 (d, 2H, *J* = 8.4Hz), 5.67 (s, 1H), 6.32 (bs, 1H), 7.45–7.53 (m, 2H), 7.66 (dt, 1H, *J*_1_ = 1.3Hz, *J*_2_ = 7.7Hz), 7.75 (dt, 1H, *J*_1_ = 1.3Hz, *J*_2_ = 7.7Hz), 8.08 (d, 1H, *J* = 1.1Hz), 8.10 (d, 1H, *J* = 1.1Hz), and 8.22–8.27 (m, 2H); ^13^C NMR (CDCl_3_, 75MHz) *δ* 46.2, 102.8, 124.5 (2C), 126.6 (2C), 128.1 (2C), 130.6, 132.6, 133.5, 135.2, 143.5, 147.7, 148.0, 181.9, and 183.2; MS (ES+): *m/z* = 309 [M+ H].

#### 4.1.3. 2-[(4-Methoxybenzyl) amino]naphthalene-1, 4-dione (8c)

The compound was prepared using a procedure similar to the one used in the preparation of compound **8a** using 1,4-naphthoquinone **7** (5.0 g, 31.62mmol) in THF (50 mL) and 4-methoxybenzylamine **6c** (6.15 mL, 47.42mmol) dissolved in MeOH (50 mL). Compound **8c** was obtained as a red solid (7.95 g, 86%); Mp: 138–141°C; ^1^H NMR (CDCl_3_, 300MHz) *δ* 3.79 (s, 3H), 4.28 (d, 2H), 5.78 (s, 1H), 6.15 (bs, 1H), 6.85–6.92 (m, 2H), 7.19–7.28 (m, 2H), 7.58 (dt, 1H, *J*_1_ = 1.5Hz, *J*_2_ = 7.6Hz), 7.71 (dt, 1H, *J*_1_ = 1.5Hz, *J*_2_ = 7.6Hz), 8.03 (dd, 1H, *J*_1_ = 1.11Hz, *J*_2_= 7.7Hz), and 8.08 (dd, 1H, *J*_1_ = 1.1Hz, *J*_2_ = 7.7 Hz); ^13^C NMR (CDCl_3_, 75MHz) *δ* 46.5, 55.5, 101.8, 114.6 (2C), 126.4, 126.5, 128.1, 129.3 (2C), 130.7, 132.2, 133.8, 134.9, 147.9, 159.7, 182.1, and 183.2; MS (ES+): *m/z* = 294 [M+ H].

#### 4.1.4. Ethyl 1-benzyl-2-methyl-4,9-dioxo-4,9-dihydro-1H-benzo[f]indole-3-carboxylate (4a)

To a solution of compound **8a** (0.080 g, 0.30mmol) and ethyl acetoacetate (0.158 g, 1.21mmol) in MeOH and CH_2_Cl_2_ (7 : 3, 10 mL), CAN (0.584 g, 1.06mmol) was added in four portions at 10min intervals. After another 10min of stirring at room temperature, TLC analysis (100% CH_2_Cl_2_) revealed the completion of the reaction. The solvents were removed *in vacuo*. Water (50 mL) was added to the residue and extracted with CH_2_Cl_2_ (3 × 30mL). The combined CH_2_Cl_2_ layer was washed with water (2×30 mL), brine (20 mL) and dried over Na_2_SO_4_. The drying agent was filtered off, and the filtrate was concentrated under reduced pressure to obtain the crude product which was purified by column chromatography over silica gel using EtOAc/hexanes (1 : 3) as eluent to furnish the product **4a** as a yellow solid (0.091 g, 80%); Mp: 157–160°C; ^1^HNMR(CDCl_3_, 300MHz) *δ* 1.44 (t, 3H, *J* = 7.2Hz), 2.40 (s, 3H), 4.43 (q, 2H, *J* = 7.2Hz), 5.81 (s, 2H), 7.06 (d, 2H, *J* = 7Hz), 7.22–7.36 (m, 3H), 7.60–7.71 (m, 2H), and 8.03–8.19 (m, 2H); ^13^C NMR (CDCl_3_, 75MHz) *δ*: 11.2, 14.4, 49.0, 61.4, 114.7, 126.2 (2C), 126.4, 126.5, 126.9, 128.0, 129.2 (2C), 130.5, 133.1, 133.4, 133.5, 134.0, 135.8, 142.5, 164.8, 176.4, and 179.6; MS (ES+): *m/z* = 374 [M+ H].

#### 4.1.5. Ethyl 1-(4-nitrobenzyl)-4,9-dihydro-2-methyl-4,9-dioxo-1H-benzo[f]indole-3-carboxylate (4b)

Compound **8b** (0.050 g, 0.16mmol) and ethyl acetoacetate (0.021 g, 0.16mmol) were dissolved in a mixture of EtOH and CH_2_Cl_2_ (7 : 3, 33 mL) by heating the solution for 15min. After the removal of heating, CAN (0.388 g, 0.71mmol) was added in four installments at 10min intervals. After 16 h of stirring at room temperature, TLC analysis (100% CH_2_Cl_2_) showed the completion of reaction. After the solvents were removed *in vacuo*, water (50 mL) was added to the residue and extracted with CH_2_Cl_2_ (3 × 30mL). The combined CH_2_Cl_2_ layer was washed with water (2×30 mL), brine (20 mL) and dried over Na_2_SO_4_. The drying agent was filtered off, and the crude product obtained was purified by column chromatography over silica gel using 100% CH_2_Cl_2_ and recrystallized with CH_2_Cl_2_/hexanes to isolate compound **4b** as a yellow solid (0.023 g, 35%); Mp: 96–98°C; ^1^H NMR (CDCl_3_, 700MHz) *δ* 1.46 (t, 3H, *J* = 7.0Hz), 2.44 (s, 3H), 4.47 (q, 2H, *J* = 7.0Hz), 5.91 (s, 2H), 7.25 (d, 2H, *J* = 3.3Hz), 7.68 (t, 1H, *J* = 3.2Hz), 7.71 (t, 1H, *J* = 3.2Hz), 8.07 (d, 1H, *J* = 3.3Hz), 8.19 (d, 1H, *J* = 3.2Hz), and 8.21 (d, 2H, *J* = 3.3Hz); ^13^C NMR (CDCl_3_, 175MHz) *δ* 11.1, 14.4, 48.6, 61.6, 115.2, 124.6 (2C), 126.5 (2C), 127.1, 127.2 (2C), 130.4, 133.1, 133.4, 133.9, 134.0, 142.0, 143.2, 147.9, 164.5, 176.6, and 179.6; MS (ES+): *m/z* = 419 [M+ H].

#### 4.1.6. Ethyl 1-(4-methoxybenzyl)-4,9-dihydro-2-methyl-4,9-dioxo-1H-benzo[f]indole-3-carboxylate (4c)

To a solution of compound **8c** (0.080 g, 0.27mmol) in a mixture of EtOH and CH_2_Cl_2_ (7 : 3, 10 mL), ethyl acetoacetate (0.036 g, 0.27mmol) was added, and the solution was charged with CAN (0.523 g, 0.96mmol) in four portions at 10min intervals and stirred at room temperature for another 10min. TLC analysis (100% CH_2_Cl_2_) indicated the reaction was complete. After the solvents were removed under reduced pressure, water (50 mL) was added to the residue. It was extracted with CH_2_Cl_2_ (3 × 30mL), washed with water (2 × 30 mL), brine (20 mL) and dried over Na_2_SO_4_. Drying agent was filtered off, and the filtrate was concentrated *in vacuo* to obtain the crude product, which was purified by column chromatography over silica gel (eluted with 100% CH_2_Cl_2_) and recrystallized with CH_2_Cl_2_/hexanes to isolate compound **4c** as a yellow solid (0.090 g, 82%); Mp: 127–129°C; ^1^H NMR (CDCl_3_, 300MHz) *δ* 1.44 (t, 3H, *J* = 7.1Hz), 2.43 (s, 3H), 3.77 (s, 3H), 4.44 (q, 2H, *J* = 7.1MHz), 5.76 (s, 2H), 6.85 (d, 2H, *J* = 8.0Hz), 7.04 (d, 2H, *J* = 8.0Hz), 7.67–7.68 (m, 2H), and 8.10–8.15 (m, 2H); ^13^CNMR (CDCl_3_, 75MHz) *δ* 11.3, 14.4, 48.6, 55.5, 61.4, 114.6 (2C), 114.7, 126.2, 126.5, 126.9, 127.9, 128.0 (2C), 130.4, 133.2, 133.5 (2C), 134.0, 142.4, 159.4, 164.8, 176.4, and 179.7; MS (ES+): *m/z* = 404 [M+ H].

#### 4.1.7. 3-Acetyl-1-benzyl-2-methyl-1H-benzo[f]indole-4,9-dione (4d)

Compound **8a** (0.080 g, 0.3mmol) and acetylacetone (0.031 g, 0.3mmol) were dissolved in a mixture of MeOH and CH_2_Cl_2_ (7:3, 10mL). The reaction mixture was charged with CAN (0.523 g, 1.06mmol) in four installments at 10 min intervals. After stirring at room temperature for another 10 min, the reaction was complete as shown by TLC (100% CH_2_Cl_2_). The solvents were removed under reduced pressure. Water (50 mL) was added to the residue and extracted with CH_2_Cl_2_ (3×30mL). The combinedCH_2_Cl_2_ layer was washed with water (2×30 mL), brine (20 mL) and dried overNa_2_SO_4_. The drying agent was filtered off, and the solvent was removed *in vacuo*. The crude product thus obtained was purified by column chromatography over silica gel using 100% CH_2_Cl_2_ and recrystallized with CH_2_Cl_2_/hexanes to isolate compound **4d** as a yellow solid (0.065 g, 62%); Mp: 203–205°C; ^1^HNMR (CDCl_3_, 300MHz) *δ* 2.35 (s, 3H), 2.73 (s, 3H), 5.81 (s, 2H), 7.06 (d, 2H), 7.22–7.36 (m, 3H), 7.62–7.69 (m, 2H), and 8.04–8.17 (m, 2H); ^13^C NMR (CDCl_3_, 75MHz) *δ* 11.1, 31.9, 49.0, 123.3, 125.5, 126.5 (3C), 126.6, 126.9, 128.0, 129.2 (2C), 130.0, 133.4, 133.6, 133.7, 135.8, 142.2, 176.3, 180.9, and 199.3; MS (ES+): *m/z* = 344 [M+ H].

#### 4.1.8. 1-(4-Nitrobenzyl)-3-acetyl-2-methyl-1H-benzo[f]indole-4,9-dione (4e)

Compound **8b** (0.080 g, 0.26mmol) and acetylacetone (0.026 g, 0.26mmol) were dissolved in a mixture of MeOH and CH_2_Cl_2_ (7 : 4, 33mL) by refluxing for 15 min. The solution was removed from heat and added with CAN (0.642 g, 1.16mmol) in four portions at 10 min intervals. After overnight stirring at room temperature, the reaction was complete as indicated by TLC analysis (100% CH_2_Cl_2_). Upon removing solvents under reduced pressure, water (50 mL) was added and extracted with CH_2_Cl_2_ (3 × 30 mL). The combined CH_2_Cl_2_ layer was washed with water (2 × 30mL), brine (20 mL) and dried over Na_2_SO_4_. After removing the drying agent, the filtrate was removed *in vacuo* to obtain the crude product, which was purified by column chromatography over silica gel (eluted with 100% CH_2_Cl_2_) and recrystallized with CH_2_Cl_2_/hexanes to isolate compound **4e** as a yellow solid (0.023 g, 23%); Mp: 205–206°C; ^1^HNMR (CDCl_3_, 300MHz) *δ* 2.38 (s, 3H), 2.76 (s, 3H), 5.91 (s, 2H), 7.24 (d, 2H, *J* = 8.1Hz), 7.60–7.90 (m, 2H), and 8.07–8.22 (m, 4H); ^13^C NMR (CDCl_3_, 75MHz) *δ* 11.1, 31.9, 48.5, 123.6, 124.5 (2C), 125.8, 126.6, 127.1, 127.2, 129.9 (2C), 133.2, 133.6, 133.7, 133.9, 141.8, 143.1, 147.8, 176.5, 180.7, and 199.1; MS (ES+): *m/z* = 389 [M+ H].

#### 4.1.9. 1-(4-Methoxybenzyl)-3-acetyl-2-methyl-1H-benzo[f]indole-4,9-dione (4f)

The compound **4f** was prepared following a procedure similar to the one used in the preparation of compound **4d** using compound **8c** (0.080 g, 0.27mmol), acetylacetone (0.0545 g, 0.54mmol), and CAN (0.523 g, 0.96mmol to obtain compound a yellow solid (0.087 g, 85%); Mp: 172–173°C; ^1^H NMR (CDCl_3_, 300MHz) *δ* 2.38 (s, 3H), 2.73 (s, 3H), 3.77 (s, 3H), 5.76 (s, 2H), 6.85 (d, 2H, *J* = 8.4Hz), 7.05 (d, 2H, *J* = 8.4Hz), 7.68–7.71 (m, 2H), and 8.12–8.17 (m, 2H); ^13^C NMR (CDCl_3_, 75MHz) *δ* 11.2, 31.9, 48.5, 55.5, 114.3, 114.5, 123.3, 125.5, 126.6, 126.9, 127.8, 128.0, 129.9 (2C), 133.4, 133.5 (2C), 133.7, 142.2, 159.4, 176.4, 180.9, and 199.4; MS(ES+): *m/z* = 374 [M+ H].

#### 4.1.10. 1-Benzyl-2-methyl-3-(phenylcarbonyl)-1H-benzo[f]indole-4,9-dione (4g)

The compound **4g** was prepared following a procedure similar to the one used in the preparation of compound **4d** using compound **8a** (0.080 g, 0.3mmol), benzoylacetone (0.050 g, 0.3mmol), and CAN (0.584 g, 1.06mmol) to afford a yellow solid (0.063 g, 51%); Mp: 213–215°C; ^1^H NMR (CDCl_3_, 400MHz) *δ* 2.19 (S, 3H), 5.77 (s, 2H), 7.05 (d, 2H, *J* = 7.3Hz), 7.16–7.30 (m, 3H), 7.35 (t, 2H, *J* = 7.3Hz), 7.44–7.59 (m, 3H), 7.81 (d, 2H, *J* = 7.6Hz), 7.88 (d, 1H, *J* = 7.2Hz), and 8.02 (d, 1H, *J* = 7.2Hz); ^13^C NMR (CDCl_3_, 100MHz) *δ* 11.2, 49.3, 121.7, 126.8 (3C), 126.9, 127.0, 127.1, 128.3, 128.9, 129.5 (2C), 129.7 (2C), 130.0 (2C), 133.6 (2C), 133.7, 134.0, 134.1, 138.7, 141.3, 176.5, 180.2, and 193.4; MS (ES+): *m/z* = 406 [M + H].

#### 4.1.11. 1-(4-Nitrobenzyl)-2-methyl-3-(phenylcarbonyl)-1H-benzo[f]indole-4,9-dione (4h)

The compound **4h** was prepared following a procedure similar to the one used in the preparation of compound **4e** using compound **8b** (0.050 g, 0.16mmol), benzoylacetone (0.026 g, 0.16mmol), and CAN (0.400 g, 0.73mmol) to furnish a yellow solid (0.028 g, 38%); Mp: 231–232°C; ^1^H NMR (CDCl_3_, 300MHz) *δ* 2.31 (s, 3H), 5.95 (s, 2H), 7.32 (d, 2H, *J* = 8.6Hz), 7.47 (t, 2H, *J* = 7.6Hz), 7.58–7.70 (m, 3H), 7.91 (d, 2H, *J* = 7.8Hz), 8.00 (d, 1H, *J* = 7.0), 8.11 (d, 1H, *J* = 7.0), and 8.25 (d, 2H, *J* = 8.4Hz); ^13^C NMR (CDCl_3_, 75MHz) *δ* 10.9, 48.6, 121.8, 124.6 (2C), 126.7, 127.0, 127.3 (3C), 128.7 (2C), 129.5, 129.7 (2C), 133.4, 133.5 (2C), 133.6, 133.8, 138.3, 140.6, 143.2, 147.8, 176.4, 179.7, and 192.8; MS (ES+): *m/z* = 451 [M+ H].

#### 4.1.12. 1-(4-Methoxybenzyl)-2-methyl-3-(phenylcarbonyl)-1Hbenzo[f]indole-4,9-dione (4i)

The compound **4i** was prepared following a procedure similar to the one used in the preparation of compound **4d** using compound **8c** (0.080 g, 0.27 mmol), benzoylacetone (0.0885 g, 0.54mmol), and CAN (0.523 g, 0.96mmol) to obtain a yellow solid (0.092 g, 91%); Mp: 233–235°C; ^1^H NMR (CDCl_3_, 300MHz) *δ* 2.31 (s, 3H), 3.80 (s, 3H), 5.80 (s, 2H), 6.87–6.91 (m, 2H), 7.12 (d, 2H, *J* = 8.4Hz), 7.45 (t, 2H, *J* = 7.7Hz), 7.55–7.70 (m, 3H), 7.88–7.91 (m, 2H), 7.99 (dd, 1H, *J*_1_ = 7.3Hz, *J*_2_ = 1.7Hz), and 8.15 (dd, 1H, *J*_1_ = 7.3Hz, *J*_2_ = 1.7Hz); ^13^C NMR (CDCl_3_, 75MHz) *δ* 11.1, 48.7, 55.5, 114.7 (2C), 121.5, 126.8 (2C), 126.9 (2C), 127.9, 128.1 (3C), 128.7 (2C), 129.5, 129.8, 133.4, 133.5 (2C), 133.9, 138.6, 141.0, 159.5, 176.4, 180.0, and 193.3; MS (ES+): *m/z* = 436 [M+ H].

#### 4.1.13. 1-Benzyl-N,N,2-trimethyl-4,9-dioxo-4,9-dihydro-1Hbenzo[f]indole-3-carboxamide (4j)

The compound **4j** was prepared following a procedure similar to the one used in the preparation of compound **4d** using compound **8a** (0.080 g, 0.3mmol), *N,N-*dimethylacetoacetamide (0.040 g, 0.3mmol), and CAN (0.584 g, 1.06mmol) to obtain a yellow solid (0.079 g, 70%); Mp: 163–164°C; ^1^H NMR (CDCl_3_, 400MHz) *δ* 2.18 (s, 3H), 2.87 (s, 3H), 3.12 (s, 3H), 5.63–5.80 (m, 2H), 7.04 (d, 2H), 7.15–7.30 (m, 3H), 7.59 (t, 2H, *J* = 3.9Hz), and 8.04 (t, 2H, *J* = 3.9Hz); ^13^C NMR (CDCl_3_, 100MHz) *δ* 11.1, 35.3, 38.5, 49.3, 118.4, 125.2 (2C), 126.8 (2C), 127.0, 128.2, 129.4 (3C), 129.9, 133.6 (2C), 134.2, 136.2, 138.8, 166.1, 176.2, and 180.8; MS (ES+): *m/z* = 373 [M+ H].

#### 4.1.14. 1-(4-Nitrobenzyl)-4,9-dihydro-N,N,2-trimethyl-4,9-dioxo-1H-benzo[f]indole-3-carboxamide (4k)

The compound **4k** was prepared following a procedure similar to the one used in the preparation of compound **4e** using compound **8b** (0.050 g, 0.16mmol), *N,N-*dimethylacetoacetamide (0.020 g, 0.16mmol), and CAN (0.400 g, 0.73mmol) to afford a yellow solid (0.021 g, 31%); Mp: 187–189°C; ^1^H NMR (CDCl_3_, 400MHz) *δ* 2.29 (s, 3H), 2.97 (s, 3H), 3.22 (s, 3H), 5.87 (s, 2H), 7.28 (d, 2H, *J* = 8.5Hz), 7.69 (d, 2H, *J* = 2.7Hz), 8.09–8.14 (m, 2H), and 8.21 (d, 2H, *J* = 8.5Hz); ^13^C NMR (CDCl_3_, 75MHz) *δ* 10.8, 35.2, 38.3, 48.6, 118.5, 124.5 (2C), 125.2, 126.7, 126.8, 127.4 (3C), 129.5, 133.4, 133.6, 133.7, 138.2, 143.2, 147.8, 165.5, 176.1, and 180.4; MS (ES+): *m/z* = 418 [M+ H].

#### 4.1.15. 1-(4-Methoxybenzyl)-4,9-dihydro-N,N,2-trimethyl-4,9-dioxo-1H-benzo[f]indole-3-carboxamide (4l)

The compound **4l** was prepared following a procedure similar to the one used in the preparation of compound **4d** using compound **8c** (0.080 g, 0.27mmol), *N,N-*dimethylacetoacetamide (0.0705 g, 0.54mmol), and CAN (0.523 g, 0.96mmol) to obtain a yellow solid (0.092 g, 84%); Mp: 224–226°C; ^1^H NMR (CDCl_3_, 300MHz) *δ* 2.27 (s, 3H), 2.93 (s, 3H), 3.19 (s, 3H), 3.75 (s, 3H), 5.63–5.77 (m, 2H), 6.83 (d, 2H, *J* = 8.7Hz), 7.01 (d, 2H, *J* = 8.7Hz), 7.62–7.68 (m, 2H), and 8.08–8.13 (m, 2H); ^13^C NMR (CDCl_3_, 75MHz) *δ* 10.9, 35.0, 38.2, 48.6, 55.4, 114.5 (2C), 118.1, 124.8, 126.5, 126.7, 127.9, 128.1 (3C), 129.5, 133.3 (2C), 134.0, 138.4, 159.3, 165.9, 175.9, and 180.5; MS (ES+): *m/z* = 403 [M+ H].

#### 4.1.16. 6-Benzylamino-1-tosyl-1H-indole-4,7-dione (10a)

To a solution of 6-methoxy-1-tosyl-1H-indole-4,7-dione **9** (1.2 g, 3.3mmol) in a mixture of MeOH and THF (1 : 1, 50 mL) at room temperature, a solution of benzyl amine **6a** (0.5 g, 5mmol) in MeOH (4 mL) was added and stirred at room temperature for 20 hours. TLC analysis (EtOAc/CHCl_3_, 1 : 1) revealed that the reaction was complete. The solvent was removed under reduced pressure to obtain the crude product as a reddish brown residue. It was purified by column chromatography over silica gel using EtOAc/CHCl_3_ (1 : 10) as eluent to furnish the pure compound **10a** (1.3 g, 88%); ^1^H NMR (CDCl_3_, 300MHz) *δ* 2.43 (s, 3H), 4.24 (d, 2H, *J* = 5.7Hz), 5.35 (s, 1H), 6.06 (bt, 1H, *J* = 5.7Hz), 6.71 (d, 1H, *J* = 3.0Hz), 7.15–7.20 (m, 2H), 7.25–7.45 (m, 4H), 7.8 (d, 1H, *J* = 3.0Hz), and 7.99 (d, 2H, *J* = 8.4Hz); ^13^C NMR (CDCl_3_, 75MHz) *δ* 21.8, 47.3, 97.5, 108.6, 127.0, 127.8 (2C), 128.2, 128.9 (2C), 129.0 (2C), 129.8 (2C), 131.3, 134.0, 134.4, 135.7, 146.2, 147.9, 170.2, and 181.4; MS (ES+) *m/z* 407 [M+ H].

#### 4.1.17. 6-(4-Nitrobenzylamino)-1-tosyl-1H-indole-4,7-dione (10b)

To a solution of 6-methoxy-1-tosyl-1H-indole-4,7-dione **9** (0.700 g, 2.16mmol) in MeOH and THF (1 : 1, 100 mL) at room temperature, a solution of 4-nitrobenzylamine hydrochloride **6b** (0.540 g, 3.24mmol) in MeOH (4 mL) and Et_3_N(0.330 g, 3.24mmol) was added and stirred for 20 hours. TLC analysis (EtOAc/CHCl_3_, 1 : 1) revealed that the reaction was complete. The solvent was removed under reduced pressure, and the reddish yellow residue was purified by column chromatography over silica gel using EtOAc/CHCl_3_ (1 : 10) as eluent to furnish pure compound **10b** (0.700 g, 70% yield); ^1^H NMR (CDCl_3_, 300MHz) *δ* 2.45 (s, 3H), 4.42 (d, 2H, *J* = 6.0Hz), 5.23 (s, 1H), 6.19 (bt, 1H, *J* = 6.0Hz), 6.72 (d, 1H, *J* = 3.0Hz), 7.37 (d, 2H, *J* = 8.4Hz), 7.42 (d, 2H, *J* = 8.7Hz), 7.82 (d, 1H, *J* = 3.0Hz), 8.01 (d, 2H, *J* = 8.4Hz), and 8.21 (d, 2H, *J* = 8.7Hz); ^13^C NMR (CDCl_3_, 75MHz) *δ* 21.8, 46.4, 98.5, 108.7, 124.2 (2C), 126.9, 128.0, 129.0 (2C), 129.8 (2C), 131.5 (2C), 133.9, 134.1, 143.2, 146.4, 147.6, 147.8, 170.1, and 181.4; MS (ES+) *m/z* 451 [M + H].

#### 4.1.18. 6-(4-Methoxybenzylamino)-1-tosyl-1H-indole-4,7-dione (10c)

To a solution of 6-methoxy-1-tosyl-1H-indole-4,7-dione **9** (1.13 g, 3.41mmol) in a mixture of MeOH and THF (1 : 1, 100mL) at room temperature, a solution of 4-methoxybenzylamine **6c** (0.70 g, 5.1mmol) in MeOH (4 mL) was added, and the reaction mixture was stirred at room temperature for 20 hours. TLC analysis (EtOAc/CHCl_3_, 1 : 1) revealed that the reaction was complete. The solvent was removed under reduced pressure, and the reddish yellow residue obtained was purified by column chromatography over silica gel using EtOAc/CHCl_3_ (1 : 10) as eluent to furnish pure compound **10c** (1.40 g, 94%); ^1^H NMR (CDCl_3_, 300MHz) *δ* 2.43 (s, 3H), 3.80 (s, 3H), 4.16 (d, 2H, *J* = 6.0Hz), 5.35 (s, 1H), 5.99 (bt, 1H, *J* = 6.0Hz), 6.72 (d, 1H, *J* = 3.0Hz), 6.87 (d, 2H, *J* = 8.7Hz), 7.18 (d, 2H, *J* = 8.7Hz), 7.34 (d, 2H, *J* = 8.4Hz), 7.80 (d, 1H, *J* = 3.0Hz), and 7.98 (d, 2H, *J* = 8.4Hz); ^13^CNMR(CDCl_3_, 75MHz) *δ* 21.8, 46.8, 55.3, 97.3, 108.6, 114.4 (2C), 126.9, 127.7, 128.9(2C), 129.2 (2C), 129.8 (2C), 131.3, 134.0, 134.4, 146.2, 147.8, 159.5, 170.2, and 181.4; MS (ES+) *m/z* 434 [M + H].

### 4.2. CAN Mediated Oxidative Cyclization for 16–19: General Procedure

To a solution of bicyclic quinone **10a–c** (1 equiv) and *β*-dicarbonyl compound (4 equiv) in MeOH and CH_2_Cl_2_ (5 : 1), CAN (4 equiv) was added in four equal portions at 10 min intervals. The reaction mixture was stirred for another 10 min at room temperature. TLC analysis (EtOAc/hexanes, 1 : 1) revealed completion of the reaction. Solvent was completely removed under reduced pressure, and the residue was dissolved in CH_2_Cl_2_ (75 mL), washed with water (3×50 mL), brine (1 × 50mL) and dried over anhydrous Na_2_SO_4_. The drying agent was filtered off, and the solvent was evaporated under reduced pressure. The crude product obtained was purified by column chromatography over silica gel using EtOAc/hexanes (1 : 10) as eluent to obtain the pure bispyrroloquinones **5a-l** in 52–71% yield.

#### 4.2.1. 1-Benzyl-2-methyl-4,8-dioxo-7-tosyl-1,4,7,8-tetrahydropyrrolo[3,2-f]indole-3-carboxylic Acid Ethyl Ester (5a)

Following the general procedure, compound **10a** (0.094 g, 0.23mmol) was treated with ethyl acetoacetate (0.12 g, 0.92mmol) and CAN (0.51 g, 0.92mmol) in anhydrous MeOH and CH_2_Cl_2_ (5 : 1, 12mL) to furnish compound **5a** (0.081 g, 68%); ^1^H NMR (CDCl_3_, 300MHz) *δ* 1.39 (t, 3H, *J* = 7.2Hz), 2.32 (s, 3H), 2.39 (s, 3H), 4.37 (q, 2H, *J* = 7.2Hz), 5.66 (s, 2H), 6.73 (d, 1H, *J* = 3.2Hz), 6.95–7.05 (m, 2H), 7.20 (d, 2H, *J* = 8.4Hz), 7.25–7.30 (m, 3H), 7.71 (d, 1H, *J* = 3.2Hz), and 7.90 (d, 2H, *J* = 8.4Hz); ^13^C NMR (CDCl_3_, 75MHz) *δ* 10.9, 14.1, 21.7, 48.5, 61.1, 108.3, 113.9, 124.4, 126.5 (2C), 127.6, 128.7 (2C), 128.9 (2C), 129.4 (3C), 129.6, 130.1, 132.6, 134.0, 135.6, 141.2, 145.7, 164.5, 167.1, and 177.0; MS (ES+) *m/z* 515 [M + H].

#### 4.2.2. 1-(4-Nitrobenzyl)-2-methyl-4,8-dioxo-7-tosyl-1,4,7,8-tetrahydropyrrolo[3,2-f]indole-3-carboxylic Acid Ethyl Ester (5b)

Following the general procedure, compound **10b** (0.050 g, 0.11mmol) was treated with ethyl acetoacetate (0.058 g, 0.44mmol) and CAN (0.24 g, 0.44mmol) in anhydrous MeOH and CH_2_Cl_2_ (5 : 1, 12mL) to furnish compound **5b** (0.037 g, 60%); ^1^H NMR (CDCl_3_, 300MHz) *δ* 1.40 (t, 3H, *J* = 7.2Hz), 2.35 (s, 3H), 2.37 (s, 3H), 4.39 (q, 2H, *J* = 7.2Hz), 5.72 (s, 2H), 6.76 (d, 1H, *J* = 3.3Hz), 7.12 (d, 2H, *J* = 8.7Hz), 7.17 (d, 2H, *J* = 8.3Hz), 7.72 (d, 1H, *J* = 3.3Hz), 7.86 (d, 2H, *J* = 8.3Hz), and 8.13 (d, 2H, *J* = 8.7Hz); ^13^C NMR (CDCl_3_, 75MHz) *δ* 10.8, 14.2, 21.6, 48.1, 61.2, 108.5, 114.4, 124.0 (2C), 124.7, 127.2 (2C), 128.9 (2C), 129.4 (3C), 129.7, 129.9, 132.9, 134.0, 140.8, 143.1, 146.1, 147.4, 164.2, 166.9, and 176.7; MS (ES+) *m/z* 562 [M+ H].

#### 4.2.3. 1-(4-Methoxybenzyl)-2-methyl-4,8-dioxo-7-tosyl-1,4,7, 8-tetrahydro-pyrrolo[3,2-f]indole-3-carboxylic Acid Ethyl Ester (5c)

Following the general procedure, compound **10c** (0.054 g, 0.12mmol) was treated with ethyl acetoacetate (0.064 g, 0.50mmol) and CAN (0.27 g, 0.50mmol) in anhydrous MeOH and CH_2_Cl_2_ (5 : 1, 12mL) to furnish compound **5c** (0.047 g, 71%); ^1^H NMR (CDCl_3_, 300MHz) *δ* 1.38 (t, 3H, *J* = 7.2Hz), 2.33 (s, 3H), 2.41 (s, 3H), 3.78 (s, 3H), 4.35 (q, 2H, *J* = 7.2Hz), 5.58 (s, 2H), 6.74 (d, 1H, *J* = 3.2Hz), 6.77 (d, 2H, *J* = 8.7Hz), 6.91 (d, 2H, *J* = 8.7Hz), 7.24 (d, 2H, *J* = 8.1Hz), 7.71 (d, 1H, *J* = 3.2Hz), and 7.93 (d, 2H, *J* = 8.1Hz); ^13^C NMR (CDCl_3_, 75MHz) *δ* 11.0, 14.2, 21.7, 48.1, 55.3, 61.0, 108.3, 114.0, 114.1 (2C), 124.5, 127.8, 128.1 (2C), 129.0 (2C), 129.4, 129.5 (2C), 129.6, 130.2, 132.6, 134.2, 141.1, 145.8, 159.1, 164.5, 167.1, and 176.9; MS (ES+) *m/z* 546 [M+ H].

#### 4.2.4. 3-Acetyl-1-benzyl-2-methyl-7-tosyl-1H,7H-pyrrolo[3,2-f]indole-4,8-dione (5d)

Following the general procedure, compound **10a** (0.050 g, 0.12mmol) was treated with acetyl acetone (0.049 g, 0.48mmol) and CAN (0.27 g, 0.48mmol) in anhydrous MeOH and CH_2_Cl_2_ (5 : 1, 12mL) to furnish compound **5d** (0.040 g, 67%); ^1^H NMR (CDCl_3_, 300MHz) *δ* 2.26 (s, 3H), 2.40 (s, 3H), 2.65 (s, 3H), 5.66 (s, 2H), 6.74 (d, 1H, *J* = 2.4Hz), 6.90–7.00 (m, 2H), 7.21 (d, 2H, *J* = 8.0Hz), 7.20–7.40 (m, 3H), 7.73 (d, 1H, *J* = 2.4Hz), and 7.93 (d, 2H, *J* = 8.0Hz); ^13^C NMR (CDCl_3_, 75MHz) *δ* 10.9, 21.8, 31.6, 48.5, 108.2, 122.6, 123.7, 126.5 (2C), 127.6, 128.7 (2C), 129.0 (2C), 129.5 (3C), 129.6, 130.1, 132.3, 133.9, 135.6, 140.8, 145.9, 167.0, 178.3, and 199.1; MS (ES+) *m/z* 487 [M+ H].

#### 4.2.5. 3-Acetyl-2-methyl-1-(4-nitrobenzyl)-7-tosyl-1H,7H-pyrrolo[3,2-f]indole-4,8-dione (5e)

Following the general procedure, compound **10b** (0.045 g, 0.10mmol) was treated with acetyl acetone (0.040 g, 0.40mmol) and CAN (0.22 g, 0.40mmol) in anhydrous MeOH and CH_2_Cl_2_ (5 : 1, 12 mL) to furnish compound **5e** (0.027 g, 58%); ^1^H NMR (CDCl_3_, 300MHz) *δ* 2.30 (s, 3H), 2.37 (s, 3H), 2.67 (s, 3H), 5.72 (d, 2H), 6.75 (d, 1H, *J* = 3.3Hz), 7.14 (d, 2H, *J* = 8.7Hz), 7.19 (d, 2H, *J* = 8.1Hz), 7.74 (d, 1H, *J* = 3.3Hz), 7.88 (d, 2H, *J* = 8.1Hz), and 8.14 (d, 2H, *J* = 8.7Hz); ^13^C NMR (CDCl_3_, 75MHz) *δ* 10.8, 21.6, 31.7, 48.0, 108.5, 122.9, 124.0, 124.1, 127.2 (2C), 128.9, 129.0 (2C), 129.4 (2C), 129.7 (2C), 129.9, 132.5, 133.7, 140.4, 143.0, 146.2, 147.4, 166.9, 178.1, and 198.8; MS (ES+) *m/z* 532 [M+H].

#### 4.2.6. 3-Acetyl-1-(4-methoxybenzyl)-2-methyl-7-tosyl-1H,7Hpyrrolo[3,2-f]indole-4,8-dione (5f)

Following the general procedure, compound **10c** (0.070 g, 0.16mmol) was treated with acetyl acetone (0.064 g, 0.64mmol) and CAN (0.35 g, 0.64mmol) in anhydrous MeOH and CH_2_Cl_2_ (5 : 1, 12 mL) to furnish compound **5f** (0.056 g, 68%); ^1^H NMR (CDCl_3_, 300MHz) *δ* 2.28 (s, 3H), 2.41 (s, 3H), 2.63 (s, 3H), 3.78 (s, 3H), 5.58 (s, 2H), 6.73 (d, 1H, *J* = 3.0Hz), 6.78 (d, 2H, *J* = 8.0Hz), 6.93 (d, 2H, *J* = 8.5Hz), 7.25 (d, 2H, *J* = 8.0Hz), 7.73 (d, 1H, *J* = 3.0Hz), and 7.95 (d, 2H, *J* = 8.5Hz); ^13^C NMR (CDCl_3_, 75MHz) *δ* 10.9, 21.7, 31.6, 48.0, 55.3, 108.2, 114.1 (2C), 122.7, 123.8, 127.7, 128.1 (2C), 129.0, 129.1 (2C), 129.5 (2C), 129.6, 130.3, 132.3, 134.1, 140.7, 145.9, 159.1, 167.1, 178.3, and 199.0; MS (ES+) *m/z* 517 [M + H].

#### 4.2.7. 3-Benzoyl-1-benzyl-2-methyl-7-tosyl-1H,7H-pyrrolo[3,2-f]indole-4,8-dione (5 g)

Following the general procedure, compound **10a** (0.10 g, 0.25mmol), 1-phenyl-2-propanone (0.16 g, 0.99mmol) and CAN (0.47 g, 0.99mmol) in anhydrous MeOH and CH_2_Cl_2_ (5 : 1, 12mL) to furnish compound **5 g** (0.090 g, 67%); ^1^HNMR(CDCl_3_, 300MHz) *δ* 2.19 (s, 3H), 2.41 (s, 3H), 5.69 (s, 2H), 6.59 (d, 1H, *J* = 2.4Hz), 6.90–7.10 (m, 2H), 7.20–7.35 (m, 5H), 7.40 (t, 2H, *J* = 7.2Hz), 7.54 (t, 1H, *J* = 7.2 Hz), 7.68 (d, 1H, *J* = 2.4Hz), 7.82 (d, 2H, *J* = 7.5Hz), and 7.95 (d, 2H, *J* = 8.1Hz); ^13^C NMR (CDCl_3_, 75MHz) *δ* 10.7, 21.8, 48.7, 108.2, 120.9, 125.1, 126.6 (2C), 127.7, 128.4 (2C), 128.7, 128.8 (2C), 128.9, 129.0 (2C), 129.2 (2C), 129.5 (2C), 130.6, 132.0, 133.1, 134.1, 135.7, 138.4, 139.7, 145.8, 167.0, 177.3, and 192.9; MS (ES+) *m/z* 549 [M+ H].

#### 4.2.8. 3-Benzoyl-2-methyl-1-(4-nitrobenzyl)-7-tosyl-1H,7Hpyrrolo[3,2-f]indole-4,8-dione (5h)

Following the general procedure, compound **10b** (0.050 g, 0.11mmol) was treated with 1-phenyl-2-propanone (0.072 g, 0.44 mmol) and CAN (0.24 g, 0.44mmol) in anhydrous MeOH and CH_2_Cl_2_ (5 : 1, 12mL) to furnish compound **5h** (0.034 g, 52%); ^1^H NMR (CDCl_3_, 300MHz) *δ* 2.23 (s, 3H), 2.39 (s, 3H), 5.76 (s, 2H), 6.61 (d, 1H, *J* = 2.4Hz), 7.15–7.25 (m, 4H), 7.43 (t, 2H, *J* = 8.0Hz), 7.57 (t, 1H, *J* = 7.6Hz), 7.69 (d, 1H, *J* = 2.4Hz), 7.82 (d, 2H, *J* = 7.6Hz), 7.90 (d, 2H, *J* = 8.0Hz), and 8.18 (d, 2H, *J* = 8.4Hz); ^13^C NMR (CDCl_3_, 75MHz) *δ* 10.6, 21.6, 48.1, 108.4, 121.1, 124.1 (2C), 125.3, 127.3 (2C), 128.4, 128.6 (2C), 129.0 (2C), 129.2 (2C), 129.4 (2C), 129.8, 130.2, 132.2, 133.3, 133.9, 138.1, 139.2, 143.1, 146.1, 147.5, 166.9, 177.0, and 192.5; MS (ES+) *m/z* 594 [M + H].

#### 4.2.9. 3-Benzoyl-1-(4-methoxybenzyl)-2-methyl-7-tosyl-1H, 7H-pyrrolo[3,2-f]indole-4,8-dione (5i)

Following the general procedure, compound **10c** (0.080 g, 0.18mmol) was treated with 1-phenyl-2-propanone (0.090 g, 0.72mmol) and CAN (0.36 g, 0.22mmol) anhydrous MeOH and CH_2_Cl_2_ (5 : 1, 12mL) to furnish compound **5i** (0.074 g, 70%); ^1^H NMR (CDCl_3_, 300MHz) *δ* 2.21 (s, 3H), 2.43 (s, 3H), 3.8 (s, 3H), 5.61 (s, 2H), 6.59 (d, 1H, *J* = 3.3Hz), 6.81 (d, 2H, *J* = 8.4Hz), 7.01 (d, 2H, *J* = 8.4Hz), 7.25–7.30 (m, 2H), 7.40 (t, 2H, *J* = 8.0Hz), 7.54 (t, 1H, *J* = 8.0Hz), 7.68 (d, 1H, *J* = 3.3Hz), 7.81 (d, 2H, *J* = 8.0Hz), and 7.97 (d, 2H, *J* = 8.0Hz); ^13^CNMR (CDCl_3_, 75MHz) *δ* 10.8, 21.8, 48.1, 55.3, 108.2, 114.1 (2C), 120.8, 125.1, 127.8, 128.2 (2C), 128.3 (2C), 128.6, 129.1 (2C), 129.2 (2C), 129.4, 129.5 (2C), 130.6, 131.9, 133.1, 134.1, 138.3, 139.7, 146.0, 159.1, 167.1, 177.3, and 192.9; MS (ES+) *m/z* 579 [M + H].

#### 4.2.10. 1-Benzyl-2-methyl-4,8-dioxo-7-tosyl-1,4,7,8-tetrahydropyrrolo[3,2-f]indole-3-carboxylic Acid Dimethylamide (5j)

Following the general procedure, compound **10a** (0.050 g, 0.12mmol) was treated with N,N-dimethylacetoacetamide (0.064 g, 0.49mmol) and CAN (0.27 g, 0.49mmol) in anhydrous MeOH and CH_2_Cl_2_ (5 : 1, 12mL) to furnish compound **5j** (0.043 g, 68%); ^1^H NMR (CDCl_3_, 300MHz) *δ* 2.15 (s, 3H), 2.41 (s, 3H), 2.88 (s, 3H), 3.13 (s, 3H), 5.55–5.75 (m, 2H), 6.70 (d, 1H, *J* = 3.0Hz), 6.90–7.10 (m, 2H), 7.20–7.30 (m, 5H), 7.71 (d, 1H, *J* = 3.0Hz), and 7.93 (d, 2H, *J* = 8.4Hz); ^13^C NMR (CDCl_3_, 75MHz) *δ* 10.6, 21.8, 34.8, 38.0, 48.6, 108.0, 117.5, 123.3, 126.7 (2C), 127.6, 128.5, 128.7 (2C), 129.0 (2C), 129.4 (3C), 131.0, 131.9, 134.0, 135.8, 137.1, 145.8, 165.5, 167.0, and 178.0; MS (ES+) *m/z* 516 [M + H].

#### 4.2.11. 2-Methyl-1-(4-nitrobenzyl)-4,8-dioxo-7-tosyl-1,4,7,8-tetrahydropyrrolo[3,2-f]indole-3-carboxylic Acid Dimethylamide (5k)

Following the typical procedure, compound **10b** (0.045 g, 0.10mmol) was treated with N,N-dimethylacetoacetamide (0.051 g, 0.40mmol) and CAN (0.22 g, 0.40mmol) in anhydrous MeOH and CH_2_Cl_2_ (5 : 1, 12mL) to furnish compound **5k** (0.031 g, 58%); ^1^H NMR (CDCl_3_, 300MHz) *δ* 2.20 (s, 3H), 2.38 (s, 3H), 2.90 (s, 3H), 3.15 (s, 3H), 5.60–5.80 (m, 2H), 6.71 (d, 1H, *J* = 3.3Hz), 7.15–7.25 (m, 4H), 7.72 (d, 1H, *J* = 3.3Hz), 7.88 (d, 2H, *J* = 8.4Hz), and 8.14 (d, 2H, *J* = 8.7Hz); ^13^C NMR (CDCl_3_, 75MHz) *δ* 10.5, 21.6, 34.9, 38.1, 48.1, 108.1, 117.9, 123.6, 124.0 (2C), 127.4, 128.4 (2C), 129.0, 129.4 (2C), 129.7 (2C), 130.4, 132.1, 133.9, 136.8, 143.1, 146.1, 147.4, 165.1, 166.7, and 177.7; MS (ES+) *m/z* 561 [M+ H].

#### 4.2.12. 1-(4-Methoxybenzyl)-2-methyl-4,8-dioxo-7-tosyl-1,4,7, 8-tetrahydro-pyrrolo[3,2-f]indole-3-carboxylic Acid Dimethylamide (5l)

Following the typical procedure, compound **10c** (0.070 g, 0.16mmol) was treated with N,N-dimethylacetoacetamide (0.083 g, 0.64mmol) and CAN (0.35 g, 0.64mmol) in anhydrous MeOH and CH_2_Cl_2_ (5 : 1, 12mL) to furnish compound **5l** (0.063 g, 68%); ^1^H NMR (CDCl_3_, 300MHz) *δ* 2.17 (s, 3H), 2.42 (s, 3H), 2.86 (s, 3H), 3.13 (s, 3H), 3.79 (s, 3H), 5.40–5.60 (m, 2H), 6.69 (d, 1H, *J* = 3.3Hz), 6.78 (d, 2H, *J* = 7.6Hz), 6.96 (d, 2H, *J* = 8.5Hz), 7.2–7.3 (m, 2H), 7.71 (d, 1H, *J* = 3.3Hz), and 7.95 (d, 2H, *J* = 8.5Hz); ^13^C NMR (CDCl_3_, 75MHz) *δ* 10.7, 21.7, 34.8, 38.0, 48.1, 55.3, 107.9, 114.1 (2C), 117.6, 123.3, 127.9 (2C), 128.3 (2C), 128.4, 129.0, 129.4 (3C), 131.0, 131.9, 134.2, 137.0, 145.8, 159.1, 165.6, 167.0, and 178.0; MS (ES+) *m/z* 546 [M+ H].

## Figures and Tables

**Table 1:** General scheme for the oxidative free radical cyclization.

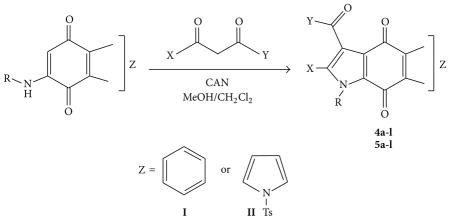				
Compd no.	R	X	Y	Z
**4a**	Benzyl	CH_3_	OCH_2_CH_3_	**I**
**4b**	4-Nitrobenzyl	CH_3_	OCH_2_CH_3_	**I**
**4c**	4-Methoxybenzyl	CH_3_	OCH_2_CH_3_	**I**
**4d**	Benzyl	CH_3_	CH_3_	**I**
**4e**	4-Nitrobenzyl	CH_3_	CH_3_	**I**
**4f**	4-Methoxybenzyl	CH_3_	CH_3_	**I**
**4g**	Benzyl	CH_3_	C_6_H_5_	**I**
**4h**	4-Nitrobenzyl	CH_3_	C_6_H_5_	**I**
**4i**	4-Methoxybenzyl	CH_3_	C_6_H_5_	**I**
**4j**	Benzyl	CH_3_	N(CH_3_)_2_	**I**
**4k**	4-Nitrobenzyl	CH_3_	N(CH_3_)_2_	**I**
**4l**	4-Methoxybenzyl	CH_3_	N(CH_3_)_2_	**I**
**5a**	Benzyl	CH_3_	OCH_2_CH_3_	**II**
**5b**	4-Nitrobenzyl	CH_3_	OCH_2_CH_3_	**II**
**5c**	4-Methoxybenzyl	CH_3_	OCH_2_CH_3_	**II**
**5d**	Benzyl	CH_3_	CH_3_	**II**
**5e**	4-Nitrobenzyl	CH_3_	CH_3_	**II**
**5f**	4-Methoxybenzyl	CH_3_	CH_3_	**II**
**5g**	Benzyl	CH_3_	C_6_H_5_	**II**
**5h**	4-Nitrobenzyl	CH_3_	C_6_H_5_	**II**
**5i**	4-Methoxybenzyl	CH_3_	C_6_H_5_	**II**
**5j**	Benzyl	CH_3_	N(CH_3_)_2_	**II**
**5k**	4-Nitrobenzyl	CH_3_	N(CH_3_)_2_	**II**
**5l**	4-Methoxybenzyl	CH_3_	N(CH_3_)_2_	**II**
